# Dental Pulp Stem Cells Differentiation Reveals New Insights in Oct4A Dynamics

**DOI:** 10.1371/journal.pone.0041774

**Published:** 2012-07-23

**Authors:** Federico Ferro, Renza Spelat, Federica D'Aurizio, Elisa Puppato, Maura Pandolfi, Antonio Paolo Beltrami, Daniela Cesselli, Giuseppe Falini, Carlo Alberto Beltrami, Francesco Curcio

**Affiliations:** 1 Department of Medical and Biological Sciences, University of Udine, Udine, Italy; 2 Department of Laboratory Medicine, University of Udine Teaching Hospital, Udine, Italy; 3 Regenerative Medicine Centre (CIME), Udine, Italy; 4 Department of Chemistry G. Ciamican, Alma Mater Studiorum University of Bologna, Bologna, Italy; Baylor College of Medicine, United States of America

## Abstract

Although the role played by the core transcription factor network, which includes c-Myc, Klf4, Nanog, and Oct4, in the maintenance of embryonic stem cell (ES) pluripotency and in the reprogramming of adult cells is well established, its persistence and function in adult stem cells are still debated. To verify its persistence and clarify the role played by these molecules in adult stem cell function, we investigated the expression pattern of embryonic and adult stem cell markers in undifferentiated and fully differentiated dental pulp stem cells (DPSC). A particular attention was devoted to the expression pattern and intracellular localization of the stemness-associated isoform A of Oct4 (Oct4A). Our data demonstrate that: *Oct4*, *Nanog*, *Klf4* and *c-Myc* are expressed in adult stem cells and, with the exception of *c-Myc*, they are significantly down-regulated following differentiation. Cell differentiation was also associated with a significant reduction in the fraction of DPSC expressing the stem cell markers CD10, CD29 and CD117. Moreover, a nuclear to cytoplasm shuttling of Oct4A was identified in differentiated cells, which was associated with Oct4A phosphorylation. The present study would highlight the importance of the post-translational modifications in DPSC stemness maintenance, by which stem cells balance self-renewal versus differentiation. Understanding and controlling these mechanisms may be of great importance for stemness maintenance and stem cells clinical use, as well as for cancer research.

## Introduction

In embryonic stem cell, Oct4 and its protein binding partners form complex autoregulatory circuits in which Oct4, Sox2, and Nanog proteins bind to each other's promoters, constituting an auto-feedback system that is necessary for embryonic stem cell self-renewal and pluripotency maintenance, by preventing differentiation [1]–[4]. It is well established that Oct4 m-RNA and protein disappear relatively quickly following differentiation [1]–[4] (although re-appearance of Oct4 m-RNA [5] and protein [6] have been noted). Differentiation pattern is complemented by a decreased expression of the proteins involved in the autoregulatory circuit, such as Nanog, Sox2 [7], and by the expression of specific lineage markers typically expressed in differentiated tissues. The transcription factor Oct4 (POU5F1) is currently considered as a main regulator of ES pluripotency and self renewal abilities [1]–[4]. Such stemness properties are attributed to Oct4A, one of the two isoforms produced by the Oct4 gene. The function of the second Oct4 isoform, Oct4B, is still largely unknown [8]. Another important difference between the two Oct4 isoforms is their intracellular localization: while Oct4A behaves as a nuclear protein, Oct4B is a cytoplasmic protein [9]. Post-translational modifications have also been reported as a regulatory mechanism for Oct4, Nanog, and Sox2 in ES cells [2], [10]–[12]. Oct4 and Sox2 are both sumoylated [11], [12], Baltus et al. [12] reported that Sox2 is acetylated [12] and also Saxe et al. [2] reported the presence of phosphorylation sites on Oct4 sequence. However, although Oct4 has been observed in adult stem cells, its functional role in this setting is still controversial [13]. In this study we used a human dental pulp stem cell (DPSC) population, whose stemness has been reported by many authors [14]–[20], to investigate and compare embryonic and adult stem cell markers in undifferentiated DPSC and afterward differentiation, with particular interest in Oct4A and its subcellular re-localization. Because of its subcellular re-localization in relation to the differentiation switch, we hypothesize here a functional role for Oct4A post-translational modifications in adult stem cells.

## Materials and Methods

### Cell isolation, culture and ethical statement

After written informed consent of donor parents and ethics approval from the Ethics Committee of the Medical Faculty of Udine, dental pulp was extracted, using a syringe needle, from human deciduous teeth of 5 to 7 year old children (n = 10) and was cultured as previously described by Ferro et al. [21]. The human serum used in this study was obtained after written informed consent of the donors from the “Medicina Trasfusionale” department of the “Santa Maria della Misericordia” hospital of Udine. Human embryonic carcinoma stem cells (Ntera2), purchased from ATCC (ATCC-LGC, Milan, IT), were used as positive control for embryonic stem markers as suggested by Liedtke et al. [22], and were cultured according to [23]. MCF7 breast cancer cell line, purchased from ATCC (ATCC-LGC, Milan, IT), was used as negative control for Oct4 expression [24]. Human osteoblast like cells, hOB, (ATCC-LGC, Milan, IT) were used as positive control for osteoblastic differentiation and were cultured by the method [25]. Human primary thyroid cells were cultured as already described by [26], and used as osteoblastic negative control. Human Supranumeral Teeth Buds (STB) were isolated, following same methods used for DPSC, cultured as reported by [27] and were used as odontoblastic positive control. HepG2, human hepato-cellular carcinoma, cell line (ATCC-LGC) was used as positive control for hepatic differentiation and was cultured as in [28]. Small fragments of cardiac tissue were used as positive control for cardiomyocyte (from discarded hearts with permission). Be2C, human neuro-blastoma (ATCC-LGC), cell line was used as positive control for neural differentiation and was cultured as reported in [29].

### Multi differentiation potential

To confirm multipotency, DPSC were differentiated toward osteoblastic, hepatic, myocytic and neural lineages as previously described [14]–[20], in a 5% CO_2_, 95% humidity incubator at 37°C with medium change twice a week, and were subjected to characterization.

### Osteoblastic differentiation

DPSCs at fifth passage in culture (P5-DPSC) were plated at a density of 4×10^4^ cells/cm^2^ and osteo-induced in a medium already described by Ferro et al. [21], [30]. One month old osteo-differentiated DPSC were used for characterization analyses except in X-Ray Diffraction (XRD) and Fourier Transform Infra Red (FTIR) spectroscopy. To demonstrate bone formation DPSC were allowed to aggregate by enzymatically detaching, approximately 5×10^6^ cells, and centrifuging them at 1×10^3^ rpm in 15 ml tube. Cells were then transferred to 100 mm dishes, covered by 2% agarose diluted 1∶1 with differentiation medium, allowed to form 3D structures and subjected to XRD and FTIR after 3 months.

### Myocyte differentiation

To induce myocyte differentiation confluent P5 DPSC were cultured in DMEM with 5% fetal calf serum (FCS); 10 ng/ml FGFb; 10 ng/ml vascular endothelial growth factor (VEGF) and 10 ng/ml IGF-1 (all from Peprotech, London, UK) [31]. Differentiation was achieved after 1 month.

### Neural differentiation

Neural differentiation proceeds in three sequential steps: specification, commitment, and differentiation [31], [32]. Neural specification, was induced by seeding DPSC (3×10^3^ cells/cm^2^) at P5 in DMEM-high glucose (Gibco-Invitrogen, Carlsbad, CA) with 10% FBS. After 24 hours, cells were shifted to a neural commitment medium and cultured for 15 days: DMEM-high glucose; 10% FBS; 5 ml/l B27 supplement (Gibco); 10 ng/ml EGF and 20 ng/ml b-FGF (both from Peprotech). Afterwards, cells were cultured for 15 days in a second neural commitment medium containing DMEM-high glucose; 5% FBS; 5 ml/l B27 supplement; 5 ng/ml EGF; 10 ng/ml b-FGF; 10 ng/ml neurotrophin-3 (NT-3); 10 ng/ml nerve growth factor (NGF); 25 ng/ml brain derived neurotrophic factor (BDNF) (all from Immunotools, Friesoythe, GE); 10 μM butylated hydroxyanisole; 25 μM 3-Isobutyl-1-methylxanthine (IBMX); 0,5 μM all trans retinoic acid and 10 nM progesterone (all from Sigma-Aldrich, St. Louis, MO). Finally, neural differentiation was achieved by exposing committed cells to a neural differentiation medium for 1 day: DMEM with 50 ng/ml BDNF; 5 μg/ml insulin; 200 μM indomethacin and 0.5 mM IBMX.

### Hepatocyte differentiation

Hepatocyte differentiation was induced in confluent DPSC at P5 in DMEM-low glucose with 1% FCS; 20 ng/ml hepatocyte growth factor (Immunotools); 10 ng/ml oncostatin; 10 mM nicotinammide; 1.25 μg/ml low density lipoprotein (LDL); 10 ng/ml fibroblast growth factor-4 (FGF-4); 4 μg/ml insulin; 1.25 g/l glucose (all from Sigma); 180 mg/l linoleic acid (MP-Biomedicals, Solon, OH) [31]. Hepatocyte differentiation was achieved after 40 days.

### Immunofluorescence (IF)

Undifferentiated and differentiated P5 DPSC, Ntera2 and MCF7 cell lines were fixed in 4% PFA. The following primary antibodies were used: Nanog (Abcam, Cambridge, MA, Cat#ab21603) 1∶125; Oct4A (Santa Cruz, Santa Cruz, CA, Cat#sc5279) 1∶150; SSEA4 (Abcam, Cat#ab16287) 1∶40; SSEA1 (Chemicon, Temecula, CA, Cat#MAB4301) 1∶40, connexin 43 (Santa Cruz, Cat#sc9059) 1∶40; cytokeratin 8 (Neomarkers, Newmarket, UK, Cat#Rb9095P0) 1∶100; cytokeratin 18 (Biogenex, Fremont, CA, Cat#AM143) 1∶20; cytokeratin 19 (Abcam, Cat#ab7754) 1∶60; β^3^-Tubulin (Abcam, Cat#ab14545) 1∶900; glyal fibrillar acid protein (Dako, Glostrup, DK, Cat#Z0334) 1∶125; neurofilament 160 kDa (Abcam, Cat#ab7794) 1∶600; osteocalcin (Abcam, Cat#ab13418) 1∶80; osteopontin (Santa Cruz, Cat#sc21742) 1∶80; serca 2 ATPase, (Abcam, Cat#ab2817) 1∶75; smooth muscle actin (Sigma, Cat#M0851) 1∶200; tyrosine hydroxylase, (Abcam, Cat#ab111) 1∶450, α-sarcomeric actin (Sigma, Cat#A2172) 1∶150. FITC-labeled anti-mouse IgG (Sigma-Aldrich, Cat#F0257) 1∶375; FITC-labeled anti-rabbit IgG (Sigma, Cat#F9887) 1∶400; TRITC-labeled anti-mouse IgM, (Jackson, Sacramento, CA, Cat#715-025-140) 1∶100, were used as secondary antibodies. Nuclear counter-staining was performed using DAPI (Pierce, Rockford, IL). Images were obtained using Leica DMI 6000B microscope connected to a Leica DFC350FX camera (Leica Microsystems).

### Real Time PCR analysis

Total RNA was extracted from undifferentiated and differentiated P5 DPSC, positive and negative control cells, using TRIzol (Gibco-Invitrogen, Carlsbad, CA). Quantitative PCR was conducted using SYBR green (Roche, Mannheim, Germany) on a 96-well-plate using Lightcycler480 (Roche). The total volume (20 µl) of each PCR reaction contained SYBR Green PCR Master Mix (Roche), 10 ng cDNA, and 0.4 µM of each of the forward and reverse primers. Real Time PCR (n = 4) was performed on undifferentiated and differentiated P5 DPSC, positive and negative controls and primer sequences, PCR product sizes, annealing temperatures and gene bank accession numbers were Oct4A 5′GTGGAGAGCAACTCCGATG 5′TGCAGAGCTTTGATGTCCTG, 121 bp, 56°C, NM_002701.4; Oct4B 5′CTGCCTTTTAAAATCCAG 5′CTGAATACCTTCCCAAAT, 151 bp, 56°C, NM_203289.3; Oct4AB, 5′CGAAAGAGAAAGCGAACCAGTAT 5′AGCCTCAAAATCCTCTCG, 213 bp, 56°C, NM_002701.4, NM_203289.3; Nanog 5′ATGCCTCACACGGAGACTGT 5′AGGGCTGTCCTGAATAAGCA, 66 bp, 56°C, NM_024865; Klf4 5′CCATCTTTCTCCACGTTCG 5′AGTCGCTTCATGTGGGAG, 109 bp, 56°C, NM_004235; c-Myc 5′GCTGCTTAGACGCTGGATTT 5′TAACGTTGAGGGGCATCG, 72 bp, 56°C, NM_002467; RNA polymerase type II (RP II) 5′GCACCACGTCCAATGACAT 5′GTGCGGCTGCTTCCATAA, 350 bp, 56°C, NM_000937. The transcript amount of each gene was normalized with the RPII. Relative fold change in expression was calculated using the ΔΔCT method (CT values <30) with respect to Ntera2 cells.

### rt-PCR analysis

Total RNA was extracted from about 2×10^6^ undifferentiated and differentiated P5 DPSC, positive and negative control cells, using TRIzol (Gibco). RNA samples were quantified by spectrophotometer. After DNase treatment (Ambion), first strand cDNA synthesis was performed with 2 μg of total RNA using 100 ng random hexa-nucleotides and 10 U/µl M-MLV reverse transcriptase (Gibco). PCR amplification was carried out in a final volume of 50 μl; using 0.1 μg cDNA; 0.2 mM dNTPs; 50 pmol of each primer (MWG operon, Ebersberg, GE) and 2 U/µl Taq I polymerase (Gibco). Tissue specific primer sequences, PCR product sizes, annealing temperatures and gene bank accession numbers are reported in Table 1. Optimal conditions and number of cycles were chosen to allow sample amplifications within PCR linear phase. Reaction products were visualized on 1–2% ethidium bromide stained gels. Semi-quantitative analysis was performed on triplicate experiments, using β-actin as normalizing gene and Quantity-One software (Bio-Rad, Hercules, CA).

**Table 1:** Tissue specific primers.

Name	5′–3′ Primer Sense	5′–3′ Primer Antisense	Length	Temp	Accession No.
Alp	GCAGGCAGGCAGCTTCAC	TCAGAACAGGACGCTCAGG	496bp	60.5°C	NM_000478.3
Osc	TCACACTCCTCGCCCTATTG	CTAGACCGGGCCGTAGAAG	293bp	58°C	NM_199173.3
Osp	CCTCCTAGGCATCACCTGTG	CCACACTATCACCTCGGCC	422bp	58°C	NM_000582.2
Osn	CACAAGCTCCACCTGGACTA	GAATCCGGTACTGTGGAAGG	525bp	58°C	NM_003118.2
BMPr-Ia	TTGCTGCATTGCTGACCTGG	CAATGCTGTGAGTCTGGAGGC	421bp	58°C	NM_004329.2
BMPr-Ib	TGCAGATATCAAAGGGACAGG	TGCAGGATTGTGAGCCCAG	649bp	58°C	NM_001203.2
BMP-2	GTGTCCCCGCGTGCTTCTTAG	ACTCCTCCGTGGGGATAGAAC	479bp	58°C	NM_001200.2
BMP-4	GCCTAGCAAGAGTGCCGTC	GCATGGTTGGTTGAGTTGAG	849bp	58°C	NM_130851.2
DSPP	AATGGGACTAAGGAAGCTG	CCATGTTGTCCTTTATCCTC	553bp	53°C	NM_014208.3
Dlx-5	CCAGTATCAGTATCACGGCG	GGATGCAGAGTTCTCCAGGT	556bp	60.5°C	NM_005221.5
Coll-I	TAAAGGGTCACCGTGGCT	CGAACCACATTGGCATCA	355bp	60°C	NM_000088.3
Cbfa-I type II	ATGCTTCATTCGCCTCACAAACAA	GGACACCTACTCTCATACTGG	987bp	60°C	NM_001015051.2
β-Actin	GCACTCTTCCAGCCTTCCTTCCTG	GGAGTACTTGCGCTCAGGAGGAGC	253bp	55°C	NM_001101.3
β_3_Tubulin	CTCAGGGGCCTTTGGACATC	CAGGCAGTCGCAGTTTTCAC	160bp	54°C	NM_006086.2
GFAP	ACCAGGACCTGCTCAATGTC	ATCTCCACGGTCTTCACCAC	199bp	60°C	NM_002055.3
Glypican	TGGGAAAGGCAAAAGCAG	CAGAAGACAGTGAGGAGGTAG	343bp	60°C	NM_001448.2
Musashi-1	CGACTCCAAAACAATTGACCC	TGAGCTTTCTTACATTCCACC	300bp	60°C	NM_002442.2
NF-H	CTGCTCAATGTCAAGATGGC	TTCTCAGGGGACTTGACTTC	484bp	54°C	NM_021076.3
NF-M	TCGTCATTTGCGCGAATACC	GCCAATTCCTCTGTAATGGC	307bp	54°C	NM_005382.2
NSE	GGGACTGAGAACAAATCCAAG	CTCCAAGGCTTCACTGTTCTC	382bp	54°C	NM_001975.2
Nopp	CCAAAGCCAAGAAAGGGAAG	'TTTATTGGGGGGAAAAAGTCAG	342bp	60°C	NM_002391.1
Vimentin	GGGACCTCTACGAGGAGGAG	CGCATTGTCAACATCCTGTC	199bp	55°C	NM_ 003380.2
ANP	GAACCAGAGGGGAGAGACAGAG	CCCTCAGCTTCCTTTTTAGGAG	406bp	64°C	NM_006172.2
Cardiac actin	CATCTATGAGGGCTACGCTTTG	AAATCCAGGGCGACATAGCACA	176bp	60°C	NM_005159.4
c-TnT	GGCAGCGGAAGAGGATGCTGAA	GAGGCAGCAAGTTGGGCATGAACGA	151bp	64°C	NM_001001432.1
Gata-4	AGACATCGCACTGACTGAGAAC	GACGGGTCACTATCTGTGCAAC	475bp	60°C	NM_002052.3
Myocardin	CATCCCCAACTTTTTCTAAGTCA	'TTCAAAGGAAGCCGAGGGCTT	250bp	60°C	NM_153604.1
Nkx-2.5	CTTCAAGCCAGAGGCCTACG	CCGCCTCTGTCTTCTTCAGC	233bp	55°C	NM_004387.2
SKMA	ACCTGGCGGGCCGCGATCT	CAGCTCGTAGCTCTTTTCCA	190bp	60°C	NM_001100.3
SMA	GCGTGGCTATTCTTCGTTACT	ATCAGGCAACTCGTAACTCTTC	148bp	60°C	NM_001613.2
α-MHC	GTCATTGCTGAAACCGAGAATG	GCAAAGTACTGGATGACACGCT	414bp	64°C	NM_002471.2
Albumin	TGAAATGGCTGACTGCTGTG	GCAGCTTTATCAGCAGCTTG	273bp	54°C	NM_000477.5
Cyp2e1	ACAGAGACCACCAGCACAACT	ATGAGCGGGGAATGACACAGA	579bp	56.5°C	NM_000773.3
Erythropoietin	CCTCTGGGCCTCCCAGTC	CCCCTGTGTACAGCTTCA	498bp	54°C	NM_000799.2
Somatostatin	CGTCAGTTTCTGCAGAAGTCC	CCATAGCCGGGTTTGAGTTA	195bp	54°C	NM_ 001048.3
Transferrin	CCAGCGGACCTGCCTAGAC	GCGTCATTCCATCCGATGG	297bp	54°C	NM_001063.2
Abcg-2	GGGTTCTCTTCTTCCTGACGACC	'TGGTTGTGAGATTGACCAACAGACC	399bp	62.5°C	NM_004827.2
c-MET	CAATGTGAGATGTCTCCAGC	CCTTGTAGATTGCAGGCAGA	559bp	56°C	NM_000245.2
Mdr-1	CAGAAACAACGCATTGCCATAGCT	TGATGATGTCTCTCACTCTGTTCC	484bp	60.5°C	NM_000927.3

### Fluorescence Activated Cell Sorting (FACS) analysis

FACS analysis was performed on undifferentiated P5 DPSC (n = 3), osteo-differentiated P5 DPSC (n = 3), hepato-differentiated P5 DPSC (n = 3), neuronal-differentiated P5 DPSC (n = 3), myo-differentiated P5 DPSC (n = 3) and Ntera2 (n = 3) cells. The following antibodies (0,1 µg/10^6^ cells) were assessed: CD10-FITC (Cat#332775); CD13-PE (Cat#347406); CD29-PE (Cat#556049); CD34-FITC (Cat#345801); CD45-FITC (Cat#345808); CD49a-PE (Cat#559596); CD73-PE (Cat#550257); CD90-PE (Cat#555596)(All from BD, San Jose, CA); CD105-PE (Serotec, Raleigh, NC, Cat#MCA1557); CD117-PE(BD, Cat#332785); CD133-PE (Miltenyi Biotec, Bergisch Gladbach, GE Cat#130-080-801); KDR-PE (R&D, Minneapolis, MN, Cat#FAB357P); VE-Cadherin (Santa Cruz, Cat#Sc9989); SSEA4(Abcam); SSEA1(Chemicon). Unconjugated antibodies for SSEA4 and SSEA1 (0.1 µg/10^6^ cells) were revealed using PE conjugated (0.02 µg/10^6^ cells) anti-mouse IgG (Chemicon, Cat#AQ326H) and Cy5 anti-mouse IgM (Jackson, Cat#715-176-020), respectively. Conjugated isotype-matching antibodies were used as negative controls. Data (20.000 events) were collected from three independent experiments using a FACS-Calibur (BD) and were expressed as mean ± standard deviations (SD).

### X-ray diffraction (XRD) and Fourier Transform Infra Red spectroscopy (FTIR)

Three month osteo-differentiated DPSC aggregates were powder reduced and subjected to XRD and FTIR. The powder X-ray diffraction patterns were recorded using a X'Celerator diffractometer (PANalytical, Almelo, NL) with Cu Kα radiation (λ = 1.5418Å) and a Ni filter in a 2 θ range between 10° and 50° using a resolution of 0.05°. Prior the collection of the diffraction data, samples have been ground in a mortar and put onto a low background silica holder. In the FTIR analysis each powdered sample (approximately 0.1 mg) was mixed with about 10 mg of anhydrous KBr. The mixtures were pressed into 7 mm diameter discs. Pure KBr discs were used as background. The analysis was performed at 4 cm^−1^ resolution using a Nicolet 380 FTIR spectrometer.

### Albumin quantification

During hepatic differentiation, medium was collected and immediately snap-frozen on day 0, 25, 26, 28, 30, 31. Albumin concentration was determined using human albumin ELISA Quantitation Kit, (Bethyl, Montgomery, TX, Cat#E80-129) following the manufacturer instructions [33]. Experiments were performed in triplicate in 96 well plates. Coating step was performed with (Bethyl, Cat#A80-129A) antibody 1∶100 in 0.05 M carbonate-bicarbonate pH 9.6, for 1 hour at RT and HRP detection antibody was performed with (Bethyl, Cat#A80-129P) 1∶40.000 in 50 mM Tris, 0.14 M NaCl, 1% BSA, 0.05% Tween-20, pH 8.0 for 1 hour at RT. Tetramethylbenzidine (TMB) substrate reaction was blocked with 2 M H_2_SO_4._ Absorbance was determined using a microtiter plate reader at 450 nm for TMB, and was converted in concentration values using the program Labsystems genesis V2.16.

### Western Blot (WB)

Whole cell extracts from P5 DPSC undifferentiated, differentiated, Ntera2 (positive) and MCF7 (negative) control cells were obtained using a lysis buffer containing 25 mM HEPES pH 7.6; 0.3M NaCl; 1.5 mM MgCl_2_; 0.2 mM EDTA; 0.5% Nonidet P40; 0.5 mM dithiothreitol; 1X protease inhibitor; 1 mM NaF and 1 mM Na ortovanadate (all from Sigma). Cytoplasmic and nuclear extracts were obtained by modification of the method of Dignam et al. [34], [35]. Proteins extracts were run on SDS-PAGE 8% and 12% for 4 hours at 4°C to improve protein separation. Membranes were reacted with primary antibodies to Oct4A 1∶1000; Nanog 1∶800; Oct4B 1∶2000 (Santa Cruz, Cat#sc8630); c-Myc 1∶1000 (Abcam, Cat#11917); Klf-4 1∶2000 (Abcam, Cat#ab26648); incubated o/n at 4°C. Mouse anti α-tubulin (Sigma, Cat#T9026) antibody was used (1∶5000) for 1 hour at RT and rabbit anti-histone H3 (BioVision, Mountain View, CA, Cat#3623-100) (1∶500) was used o/n at 4°C to demonstrate equal protein loading and nuclear protein enrichment after nuclear-cytoplasmic extraction. Primary antibodies were reacted with HRP conjugated anti-mouse IgG (Pierce, Cat#32430), anti-rabbit IgG (Pierce, Cat#32460), both 1∶4000, and anti-goat IgG (Thermo scientific, Waltham, MA, Cat#PA1-86326) 1∶7000 for 1 hour at RT; antibody/antigen complexes were detected using advance chemiluminescence ECL Plus (GE Healthcare, Milano, IT). Semi-quantitative analyses were performed on triplicate experiments.

### Immunoprecipitation (IP)

Total cell extracts (n = 3) of 40×10^6^ undifferentiated DPSC, Ntera2 and MCF7 cells were subjected to immunoprecipitation (IP). After a pre-clearing step with Protein A/G agarose beads (SantaCruz, Cat#sc2003) for 3 hours at 4°C, lysates were incubated with protein A/G conjugated anti-Oct4A antibody (SantaCruz, Cat#sc5279-AC) o/n at 4°C. The immunocomplexes were pelleted at 4°C, subjected to 12% SDS-PAGE and were reacted with p-serine (Sigma, Cat#P3430) 1∶3000, p-tyrosine (Santa Cruz, Cat#sc7020) 1/3000, incubated o/n at 4°C to identify phosphorylated residues. Primary antibodies were reacted with HRP conjugated anti-mouse IgG and anti-rabbit IgG, both 1∶7000, for 1 hour at RT; antibody/antigen complexes were detected using ECL advance (GE Healthcare, Milano, IT). Whole undifferentiated DPSC cell extracts were used as input and were reacted with Oct4A antibody as previously described.

### Protein identification and phospho-analysis

Immunoprecipitated Oct4A samples, obtained from undifferentiated DPSC, were subjected to 12% SDS-PAGE and stained by MS compatible FireSilver Staining Kit (Proteome factory, Berlin, GE). The bands corresponding to those obtained in WB were excised, destained and washed for 1 h in dH2O. Proteins were allowed to diffuse out of the crushed gel overnight at 30°C, by incubation in 0.5 ml of elution buffer (50 mM Tris-HCl, 150 mM NaCl, and 0.1 mM EDTA; pH 7.5), and supernatants were recovered after centrifugation at 10.000× g for 10 minutes [36]. Then samples were reduced, alkylated with iodoacetamide, and digested with trypsin (Sigma) at 20 ng/μl and 37°C for 90 min. The resulting peptides were concentrated on a ZipTip_C18_ (Millipore) micropurification column, and eluted onto an anchorchip target for analysis on a Bruker Autoflex III MALDI TOF/TOF instrument (Bruker Daltonik, Bremen, GE). The peptide mixture was analyzed in positive reflector mode for accurate peptide mass determination. MALDI MS/MS was performed approximately 12 peptides for partial peptide sequencing and the MS and MS/MS spectra were combined and used for database searching using the MASCOT software.

Separately, Oct4A higher band derived from undifferentiated DPSC was electrophoresed into 12% SDS-PAGE gel, excised and processed as previously reported. Then Oct4A samples and phospho protein standard were digested with Glu-C at 10 ng/μl (Sigma), diluted in 50 mM Tris buffer for 2 hours at 37°C. Samples were also purified on TiO_2_ POROS R3 micro-column (Glygen Corp, Columbia, MD), the amount purified was ∼2 pmol of the standard and ∼100 pmol of the samples. Finally, elutes were used for detection by mass spectrometry and the MS and MS/MS spectra were combined and used for database searching using the MASCOT software.

### Statistical analysis

Data from experiments are expressed as mean ± SD of at least three independent experiments. Statistical significance was determined by unpaired Student's *t* test; *P*<0.05 was considered significant.

## Results

### Primary cells isolation and differentiation

**Figure 1:**
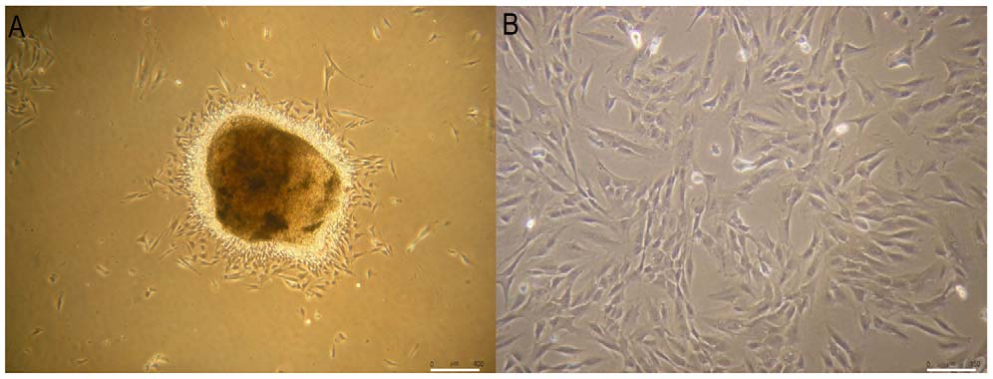
Undifferentiated DPSC morphology. (A) After 5 days of culture, we observed a robust exodus of fibroblast-like cells from the dental pulp fragments, bar scale 400 μm. (B) After 2 weeks in our expansion/selection medium, isolated DPSC were morphologically uniform and highly proliferative, bar scales 150 μm.

DPSC primary cultures were initiated from dental pulp by primary explant technique and two weeks after plating were highly proliferative and exhibited a homogeneous, fibroblastoid morphology (Fig. 1A, 1B). To demonstrate multilineage differentiation potential, DPSC at P5 were differentiated toward osteoblastic, hepatic, myocytic and neural lineages and were subjected to characterization [16]–[20], [31], [32].

**Figure 2:**
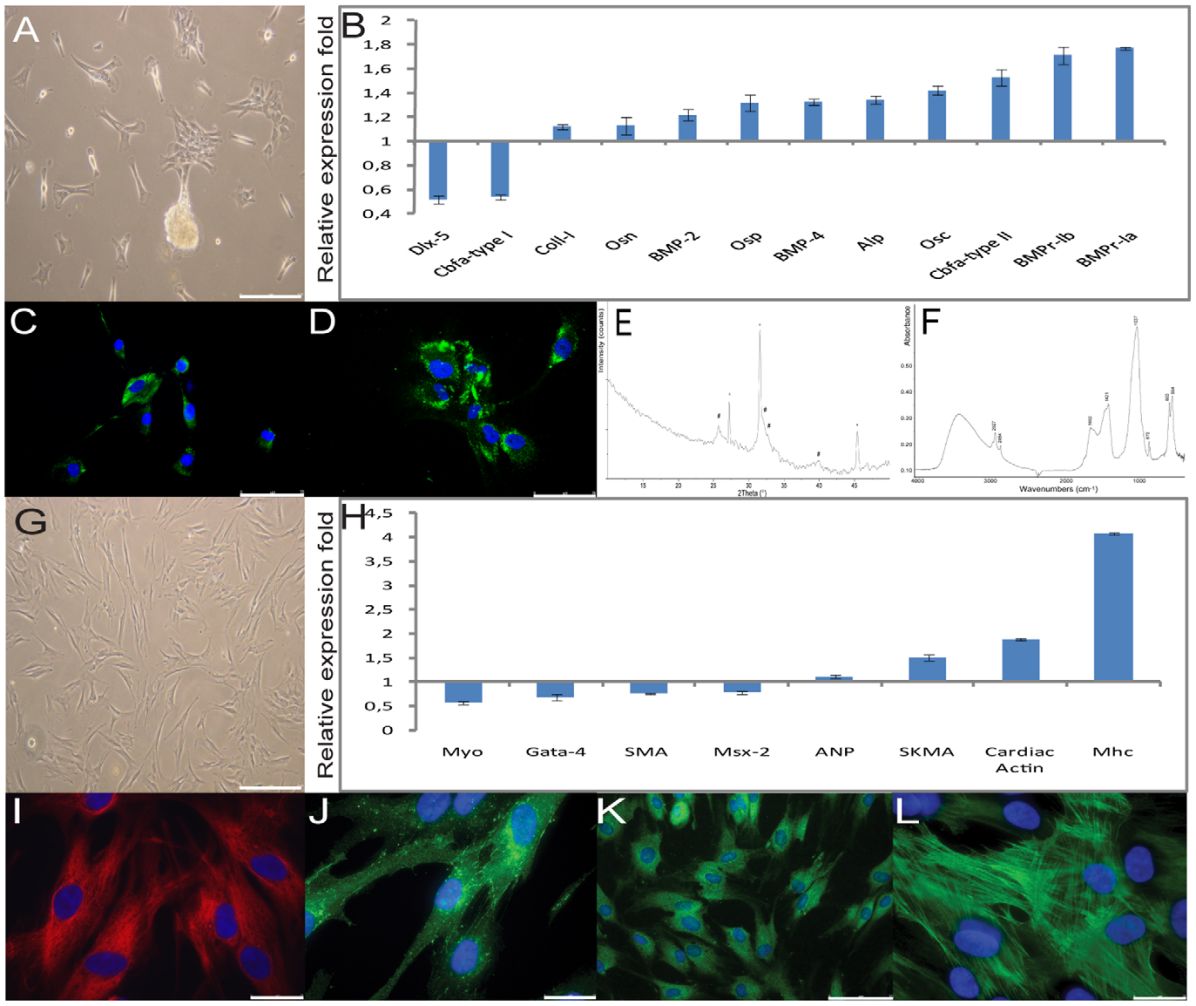
DPSC osteoblastic and myocytic differentiation characterization. (A) Evidence of the osteo-differentiated DPSC morphology change, bar scales 150 μm. (B) Semiquantitative rt-PCR was used to determine the gene expression profiles ratio of osteoblastic specific markers in 1 month osteoblastic-differentiated over undifferentiated cells, relative to housekeeping genes. Values were expressed as mean ± SD and were normalized using β-actin. X-axis shows relative expression fold Y-axis tested markers, (adjusted *P*<0.05). (C) FITC labeled antibody was used to evaluate, by IF, osteocalcin expression in 1 month osteoblastic-differentiated DPSC, bar scales 75 μm. (D) FITC labeled antibody was used to evaluate, by IF, ostepontin expression in 1 month osteoblastic-differentiated DPSC, bar scales 75 μm. Nuclei were counter stained with DAPI. (E) X-ray powder diffraction patterns from the DPSC aggregates after 3 months of osteoblastic induction. The main diffraction peaks of carbonate hydroxyapatite (#) and sodium chloride (°) and the wave numbers of the absorption peaks are indicated. (F) The FTIR spectrum shows absorption peaks at the characteristic peaks of phosphate group (ν^3^ = 1037 cm-1; ν^4^ = 603 cm^−1^ and 564 cm^−1^) and carbonate group (ν^3^ = 1421 cm^−1^). (G) Evidence of the myocyte-differentiated DPSC morphology change, bar scales 150 μm. (H) Semiquantitative rt-PCR was used to determine the gene expression profiles ratio of myocytic specific markers in 1 month myocyte-differentiated over undifferentiated DPSC cells. Values were expressed as mean ± SD and were normalized using β-actin. X-axis shows relative expression fold Y-axis tested markers, (adjusted *P*<0.05). (I) TRITC labeled antibody was used to evaluate, by IF, sarcomeric actin expression in 1 month myocyte-differentiated DPSC, bar scales 25 μm. (J) FITC labeled antibody was used to evaluate, by IF, connexin 43 expression in 1 month myocyte-differentiated DPSC, bar scales 25 μm. (K) FITC labeled antibody was used to evaluate, by IF, ATPase pump serca 2 expression in 1 month myocyte-differentiated DPSC, bar scales 75 μm. L: FITC labeled antibody was used to evaluate, by IF, smooth muscle actin expression in 1 month myocyte-differentiated DPSC, bar scales 25 μm. Nuclei were counter stained with DAPI.

Then DPSC at P5 were exposed for 1 month to differentiation conditions to induce osteoblastic phenotype [37]–[39], during this period DPSC changed their morphology developing an asymmetric shape with an enlarged end (Fig. 2A). Subsequently, cells were analyzed by rt-PCR to evidence the expression of the osteo-specific markers: Cbfa-1; alkaline phosphatase (Alp); Bone Morphonetic Proteins (BMPs), (Coll-I); osteocalcin (Osc), osteonectin (Osn) and osteopontin (Osp), distal less homeobox (Dlx-5), and moreover Dentin SialoPhosphoProtein (DSPP) [30]. Semiquantitative rt-PCR on osteo-differentiated over undifferentiated DPSC showed up-regulation of Cbfa-I type II (1.53±0.069) fold, Alp (1.34±0.033) fold, Osn (1.13±0.07) fold, Coll-I (1.12±0.021) fold, Osc (1.42±0.038) fold, Osp (1.32±0.067) fold, BMP-2 (1.24±0.045) fold, BMP4 (1.33±0.025) fold, BMPr-Ia (1.77±0.015) fold and BMP-Ib (1.71±0.07) fold in differentiated cells. Whereas Cbfa-1 type I and Dlx-5 expression were down-regulated during differentiation (0.54±0.023) and (0.52±0.034) fold respectively (Fig. 2B). Instead DSPP m-RNAs were not expressed by undifferentiated or differentiated cells (Data not shown) [39]. Osteocalcin (Fig. 2C), a late osteoblastic marker, as well as the early osteoblastic differentiation marker, osteopontin (Fig. 2D) were expressed after induction as shown by IF [30]. We used XRD and FTIR to evaluate the presence of hydroxyapatite (HA) in three month osteo-differentiated DPSC aggregates. XRD diffraction pattern shows the typical diffraction peaks of HA around 25.8° and 32° of 2θ and sodium chloride at 27.3°, 31.6° and 45.4° of 2θ (Fig. 2E) [21], [30]. The FTIR spectrum (Fig. 2F) shows absorption peaks characteristic of phosphate group (ν^3^ = 1037 cm-1; ν^4^ = 603 cm^−1^ and 564 cm^−1^) and carbonate group (ν^3^ = 1421 cm-1) [21], [30].

Cardiomyocyte differentiation was shown after 4 weeks in differentiation, during this period DPSC changed morphology to become elongated and irregular (Fig. 2G). Semiquantitative rt-PCR evidenced up-regulation for atrial natriuretic peptide (ANP) (1.11±0.034) fold, skeletal muscle actin (SKMA) (1.51±0.065) fold, cardiac actin (CA) (1.88±0.021) fold and myosin heavy chain (Mhc) (4.08±0.016) fold gene expression, while messenger expression for GATA-4 binding protein (GATA-4) (0.68±0.07) fold; smooth muscle actin (SMA) (0.76±0.012) fold, cardiac specific homeobox protein (Csx/Nkx-2.5) (0.78±0.034) fold and myocardin (Myo) (0.57±0.03) fold, decreased in differentiation as demonstrated by semiquantitative rt-PCR (Fig. 2H).

Differentiated DPSC showed organized filaments for α-sarcomeric actin (Fig. 2I) and gap-junctions presence was demonstrated by connexin-43 (Cx-43) in proximity of contact sites (Fig. 2J). Serca 2 ATPase pump (Fig. 2K) was also identified in differentiated cells. α-smooth muscle actin (SMA) was highly expressed during differentiation showing a specific filamentous structure as demonstrated by IF (Fig. 2L). It has been reported that SMA is present in embryonic or fetal but not in adult cardiomyocytes, suggesting that these phenotype may represent an early differentiation stage [40], [41].

**Figure 3:**
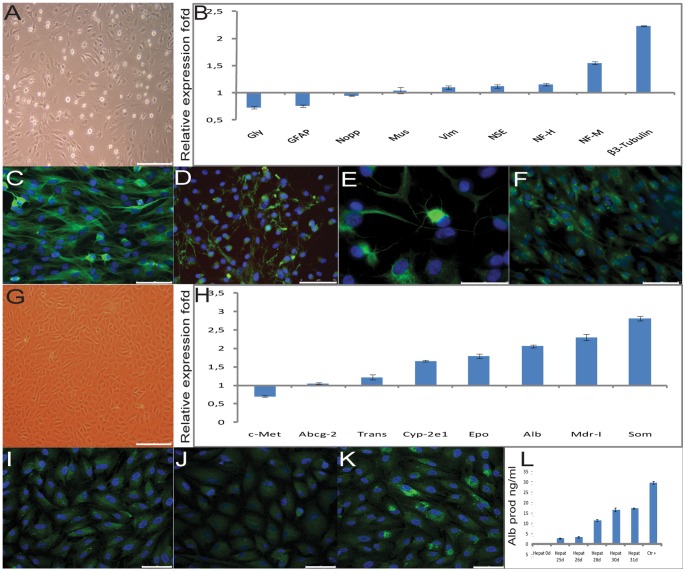
DPSC neuronal and hepato differentiation characterization. (A) Evidence of the neuronal-differentiated DPSC morphology change, bar scales 150 μm. (B) Semiquantitative rt-PCR was used to determine the gene expression profiles ratio of neuronal specific markers in 1 month neuronal-differentiated over undifferentiated DPSC cells. Values were expressed as mean ± SD and were normalized using the β-actin. X-axis shows relative expression fold Y-axis tested markers, (adjusted *P*<0.05). (C) FITC labeled antibody was used to evaluate, by IF, β^3^-tubulin expression in 1 month neuronal-differentiated DPSC, bar scales 75 μm. (D) FITC labeled antibody was used to evaluate, by IF, tyrosine hydroxylase expression in 1 month neuronal-differentiated DPSC, bar scales 50 μm. (E) FITC labeled antibody was used to evaluate, by IF, neurofilament-medium expression in 1 month neuronal-differentiated DPSC, bar scales 25 μm. (F) FITC labeled antibody was used to evaluate, by IF, glyal fibrillar acid protein expression in 1 month neuronal-differentiated DPSC, bar scales 75 μm. Nuclei were counter stained with DAPI. (G) Evidence of the hepato-differentiated DPSC morphology change, bar scales 150 μm. (H) Semiquantitative rt-PCR was used to determine the gene expression profiles ratio of hepatic specific markers in 40 days hepato-differentiated over undifferentiated DPSC cells. Values were expressed as mean ± SD and were normalized using the β-actin. X-axis shows relative expression fold Y-axis tested markers, (adjusted *P*<0.05). (I) FITC labeled antibody was used to evaluate, by IF, cytokeratin 8 expression in 1 month hepato-differentiated DPSC, bar scales 75 μm. (J) FITC labeled antibody was used to evaluate, by IF, cytokeratin 18 expression in 1 month hepato-differentiated DPSC, bar scales 75 μm. (K) FITC labeled antibody was used to evaluate, by IF, cytokeratin 19 expression in 1 month hepato-differentiated DPSC, bar scales 75 μm. Nuclei were counter stained with DAPI. L: ELISA assay showed a time dependent increased albumin secretion in the medium from 11 ng/ml on day 25 to 25 ng/ml on day 31 after hepatic differentiation. Values were expressed as mean ± SD of three independent experiments, X-axis secreted albumin concentration (ng/ml) Y-differentiation days, (adjusted *P*<0.05).

Morphology of neural-differentiated DPSC closely resembled mature neurons: they had a large number of neurites, increased 3 to 4 weeks after differentiation, with significant branching (Fig. 3A) [42]. Semiquantitative rt-PCR showed that DPSC increased m-RNA expression for ß3-Tubulin (ß3-Tubulin) (2.23±0.01) fold, neurofilament-medium (NF-M) (1.55±0.023) fold, neurofilament-heavy (NF-H) (1.15±0.021) fold, vimentin (Vim) (1.1±0.027) fold, musashi homolog 1 (Mus) (1.04±0.058) fold. Instead, neuronal specific enolase (NSE) (1.12±0.034) fold, glial fibrillar acidic protein, (GFAP) (0.76±0.019) fold, neurite growth promoting protein (Nopp) (0.95±0.01) fold and glypican (Gly) (0.73±0.023) fold, expression decreased as demonstrated by semiquantitative rt-PCR (Fig. 3B). ß3-Tubulin was largely expressed in DPSC, showing a typical filamentous expression pattern, after differentiation as shown by immunofluorescence (Fig. 3C) and at the same time neuro-differentiated DPSC expressed tyrosine hydroxylase along neurites (Fig. 3D). Structural neurofilaments NF-M were expressed only during differentiation, the positivity was about 50% (Fig. 3E) [32]. Consistently with the evidence [43], which proves that GFAP and ß3-Tubulin are co-expressed during early differentiation phase, GFAP (Fig. 3F), an astrocyte marker, was always expressed in DPSC but afterward differentiation was less expressed and organized.

One month after hepatic-differentiation, DPSC assumed a small globular shape (Fig. 3G) and increased, with respect to undifferentiated cells, the expression of albumin (Alb) (2.06±0.035) fold, transferrin (Trans) (1.22±0.065) fold, somatostatin (Soma) (2.81±0.061) fold, erythropoietin (Epo) (1.79±0.056) fold, multidrug resistence protein-I (MDR-I) (2.3±0.079) fold, cytochrome P-450 subunit 2e1 (Cyp 2e1) (1.66±0.023) fold, expressed similar quantities for ATP binding cassette-2 (Abcg-2) (1.05±0.023) fold and decreased the expression of hepatocyte growth factor receptor (c-MET/HGF-r) (0.7±0.02) fold, as demonstrated by semiquantitative rt-PCR (Fig. 3H) [44]. By immunofluorescence, cells were positive for the epithelial hepato-specific cytokeratins 8, 18 and 19 (Fig. 3I, 3J, 3K) only after differentiation showing a homogeneous punctate organization. Finally, they acquired hepatic function such as albumin production and secretion, which increased from (11±0.2 ng/ml) on day 25 to (25±0.1 ng/ml) on day 31 compared with positive control included in ELISA assay (29±0.5 ng/ml) (Fig. 3L).

### ES and adult stem cell markers expression during differentiation

**Figure 4:**
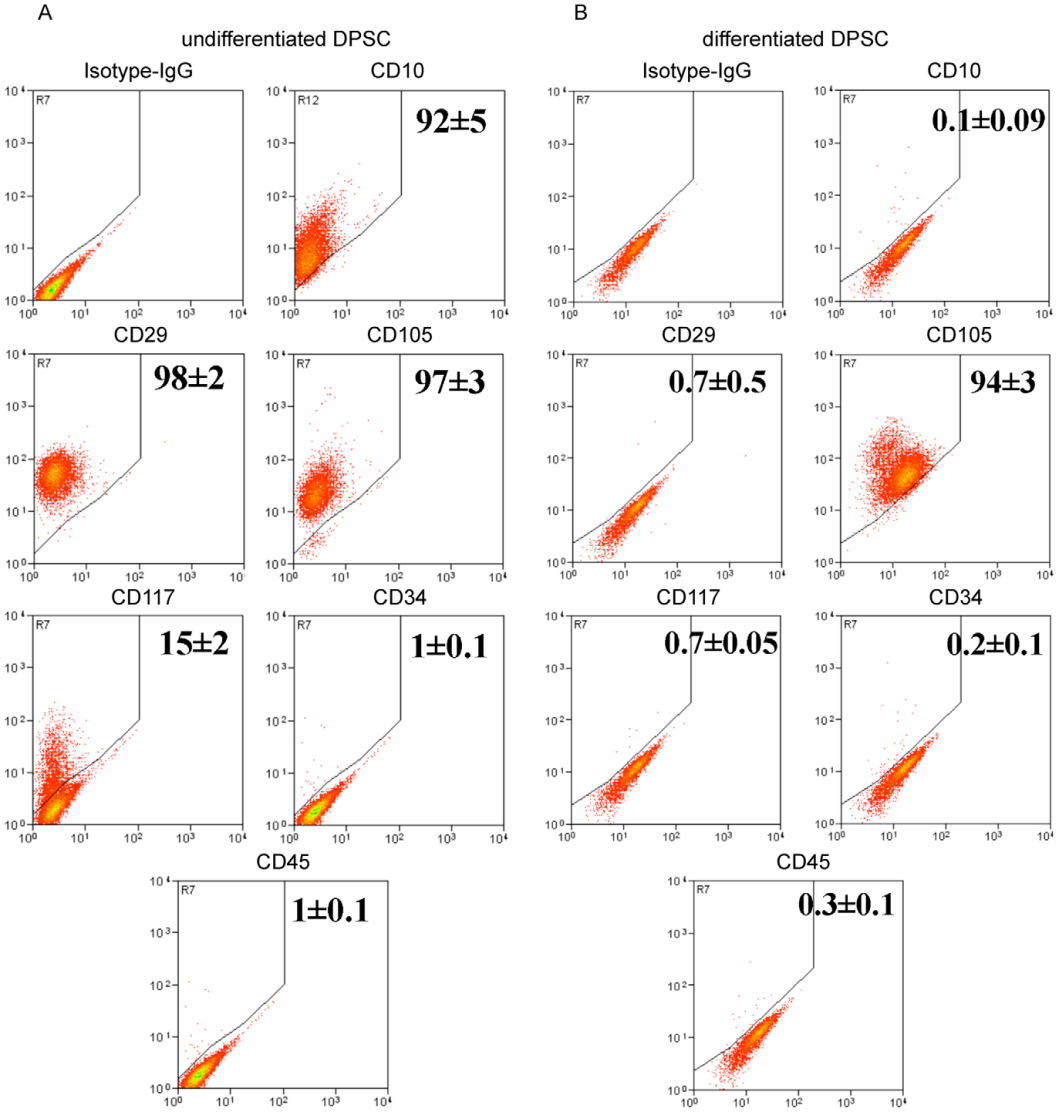
Cluster differentiation surface markers analysis. (A, B) CDs Representative Flow Cytometry for dot plots of undifferentiated and osteo-differentiated DPSC. Gate was defined considering the isotype control IgG-stained sample versus specific antibody staining profile. Results reported in each plot, X-axis shows relative fluorescence Y-axis the number of events, indicate the percentage of positive cells (mean ± standard deviation) of n = 3 experiments, (adjusted *P*<0.05).

**Figure 5:**
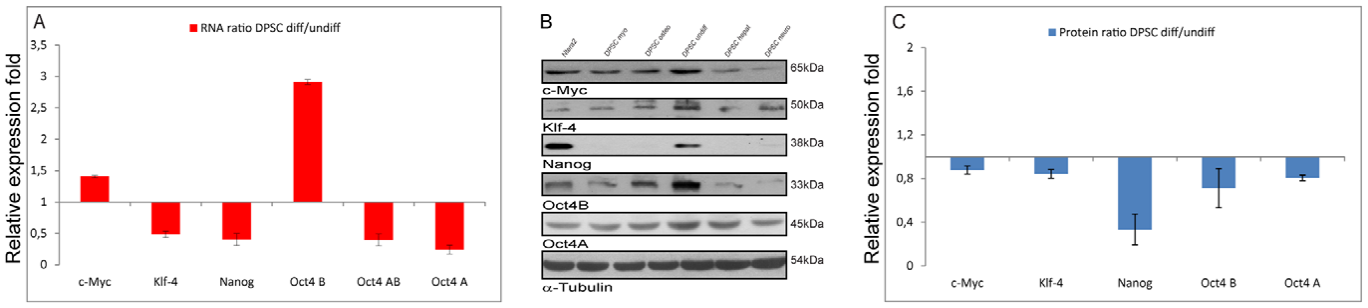
Embryonic stem cell markers 1. (A) The graph shows c-Myc, Klf-4; Nanog; Oct4B; Oct4AB; Oct4A gene expression profiles in differentiated over undifferentiated DPSC cells, relative to the housekeeping gene RNA polymerase type II. Data are averages and standard deviations of three independent samples, X-axis shows relative expression fold Y-axis tested markers, (adjusted *P*<0.05). (B) Ntera2, differentiated and undifferentiated DPSC, whole cell extracts were reacted with anti-c-Myc, Klf-4, Nanog, Oct4B, Oct4A antibodies. Lane 1: Ntera2; Lane 2: myo-differentiated DPSC; Lane 3: osteo-differentiated DPSC; Lane 4: undifferentiated DPSC; Lane 5: hepato-differentiated DPSC, Lane 6: neuro-differentiated DPSC. (C) Graph evidences DPSC differentiated over undifferentiated protein expression ratio for c-Myc, Klf-4, Nanog, Oct4B, Oct4A. Data are expressed as mean ratio ± SD of three independent experiments and were normalized using α-Tubulin, X-axis shows relative expression fold Y-axis tested markers.

To establish whether differentiation of P5 DPSC was associated with changes in the expression of cluster differentiation (CDs) markers, which were previously demonstrated to be expressed on human mesenchymal stem cells [16]–[20], [31], [32], [45]–[48], FACS analysis were performed. Results evidenced that differentiation process was associated with a significant reduction (p<0.05) in the expression of CD10 (from 92±5% to 0.1±0.09%), CD29 (from 98±2% to 0.7±0.5) and CD117 (from 15±2% to 0.7±0.05%), while only small differences were detected for the other tested antigens which showed a general decreased expression after differentiation had taken place (Fig. 4A, 4B and Table 2). In addition we also analyzed whether P5 DPSC differentiation was associated with changes in the expression of stemness-related genes, a Real Time PCR was performed quantifying the transcripts of Oct4A, Oct4AB, Oct4B, Nanog, Klf4 and c-Myc with respect to the housekeeping gene RNA polymerase type II (RP II) and expressing the results as a ratio over undifferentiated cells (Fig. 5A). Interestingly, differentiated DPSC expressed, with respect to the undifferentiated cells, significantly reduced level of Oct4A (0.24±0.072; p<0.05) fold, Oct4AB (0.39±0.094; p<0.05) fold, Nanog (0.40±0.094; p<0.05) fold and Klf4 (0.48±0.048; p<0.05) fold. Conversely, the differentiation process was associated with a significant increase in Oct4B transcripts (2.91±0.04; p<0.05) fold and c-Myc transcripts (1.40±0.019203; p<0.05) fold.

**Table 2:** Cluster differentiation markers FACS analysis.

Marker	Undifferentiated	Osteo-Differentiated	Hepato-Differentiated	Myo-Differentiated	Neuro-Differentiated
CD10	92±5%	0.1±0.09%	0.3±0.07%	2±0.9%	0.4±0.1%
CD13	95±5%	99±1%	99±1%	95±4%	99±1%
CD29	98±2%	0.7±0.5%	1.7±0.9%	0.6±0.7%	2.1±0.2%
CD34	1±0.1%	0.2±0.1%	0.4±0.2%	1.1±0.3%	0.2±0.6%
CD45	1±0.1%	0.3±0.1%	0.9±0.2%	1.3±1%	0.3±0.09%
CD49a	97±3%	98±2%	98±0.8%	96±4%	95±2%
CD73	99±1%	99±1%	95±3%	95±4%	99±1%
CD90	99±1%	99±1%	99±1%	98±2%	97±1%
CD105	97±3%	94±3%	98±2%	94±5%	90±3%
CD117	15±2%	0.7±0.05%	1.5±0.2%	1.4±0.5%	0.5±0.09%
CD133	2±0.1%	1±0.5%	0.4±0.5%	0.6±0.4%	1±0.7%
KDR	4±0.5%	4±0.5%	5±0.5%	2±1%	6±5%
VE-Cad	1±0.1%	0.4±0.04%	1±0.3%	4±2%	0.7±0.04%

**Figure 6:**
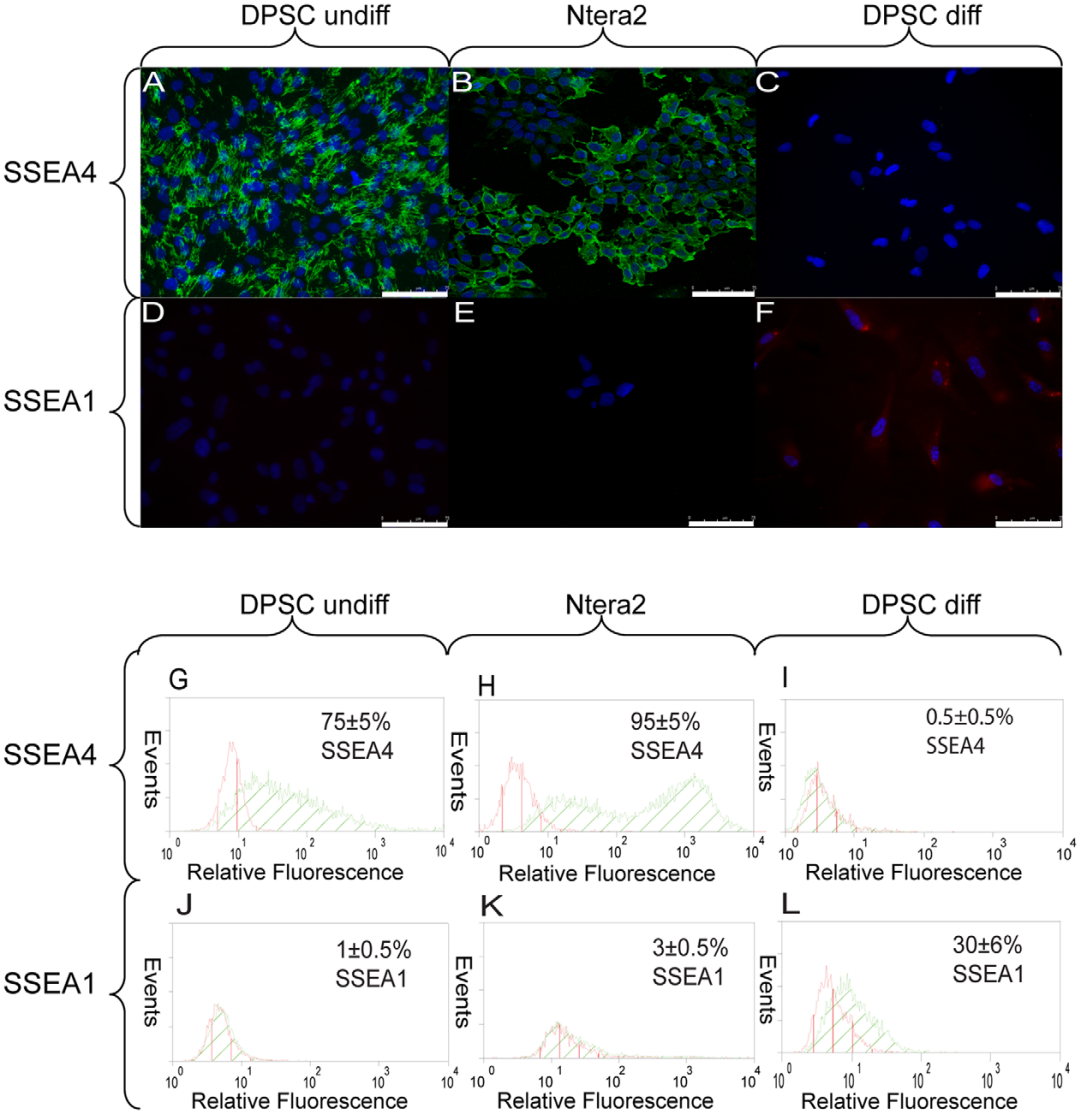
Embryonic stem cell markers 2. (A–L) FITC labeled antibody was used to evaluate, by IF, SSEA4 expression in undifferentiated DPSC (A), Ntera2 (B) and osteo-differentiated DPSC (C). TRITC labeled antibody was used to evaluate, by IF, SSEA1 expression in undifferentiated DPSC (D), Ntera2 (E), and osteo-differentiated DPSC (F). Scale bars 75 μm. Nuclei were counter-stained with DAPI. At the same time FITC labeled antibody was also used to confirm, by FACS, SSEA4 expression in undifferentiated DPSC (G), Ntera2 (H) and osteo-differentiated DPSC (I). TRITC labeled antibody was also used to confirm, by FACS, SSEA1 expression in undifferentiated DPSC (J), Ntera2 (K), and osteo-differentiated DPSC (L). X-axis shows relative fluorescence Y-axis the number of events, indicate the percentage of positive cells (mean ± standard deviation) of n = 3 experiments, (adjusted *P*<0.05).

Then WB analysis performed on undifferentiated, differentiated DPSC and Ntera2 cells [22], [23] evidenced that differentiation down-regulate the expression of Oct4A (0.80±0.026; *p*<0.05) fold, Nanog (0.33±0.14; *p*<0.05) fold, Klf4 (0.84±0.041; p<0.05) fold, Oct4B (0.71±0.18; p<0.05) fold and c-Myc (0.87±0.037; p<0.05) fold (Fig. 5B, 5C). Analyses also demonstrated that stemness associated marker SSEA4 was expressed in undifferentiated DPSC (75±5%) (Fig. 6A, 6G and Table 3) and Ntera2 cells (95±5%) (Fig. 6B, 6H and Table 3) as assessed by immunofluorescence and confirmed by FACS, while it was down-regulated in differentiated DPSC (0.5±0.5%) (Fig. 6C, 6I and Table 3). Similarly to what described for human embryonic stem cells, SSEA1 protein, (Fig. 6D, 6J and Table 3) was expressed by a low percentage of undifferentiated DPSC (1±0.5%) and Ntera2 cells (3±0.5%) (Fig. 6E, 6K and Table 3), increasing its expression after differentiation (30±6%) (Fig. 6F, 6L and Table 3).

**Table 3:** SSEA4, 1 FACS analysis.

Marker	Undifferentiated	Ntera2	Osteo- Differentiated	Hepato- Differentiated	Myo-Differentiated	Neuro-Differentiated
SSEA4	75±5%	95±5%	0.5±0.5%	0.4±0.08%	0.7±0.2%	1.5±0.3%
SSEA1	1±0.5%	3±0.5%	30±6%	35±8%	20±6%	25±10%

**Figure 7:**
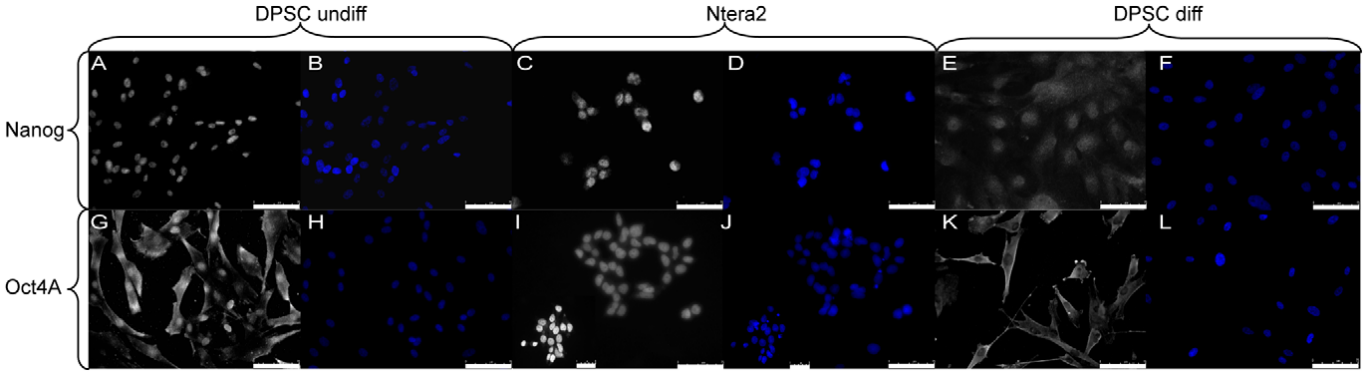
Embryonic stem cell markers 3. FITC labeled antibody was used to evidence, by IF, Nanog expression in undifferentiated DPSC (A, B), Ntera2 (C, D), and osteo-differentiated DPSC (E, F), scale bars 75 μm. In addition FITC labeled antibody was used to evidence, by IF, Oct4A expression in undifferentiated DPSC (G, H), Ntera2 (I, J), and osteo-differentiated DPSC (K, L), scale bars 50–75 μm. Nuclei were counter-stained with DAPI.

Characterization was further improved by detecting Nanog and Oct4A proteins by means of immunofluorescence. Both undifferentiated DPSC (Fig. 7A, 7B) and Ntera2 (Fig. 7C, 7D) cells showed high nuclear Nanog protein presence. By contrast, differentiated DPSC showed reduced cytoplasmic and nuclear Nanog expression (Fig. 7E, 7F). Evaluating the expression of the Oct4A protein, it was apparent that in undifferentiated DPSC Oct4A was localized not only in the nucleus but also in the cytoplasm (Fig. 7G, 7H). Conversely, Ntera2 showed a predominantly nuclear positivity and a weak cytoplasmic signal, probably because of the high nuclear to cytoplasmic ratio of these cells (Fig. 7I, 7J). In differentiated DPSC, the localization of the pluripotent-state specific transcription factor Oct4A was mainly cytoplasmic (Fig. 7K, 7L).

### Oct4A protein analysis

**Figure 8:**
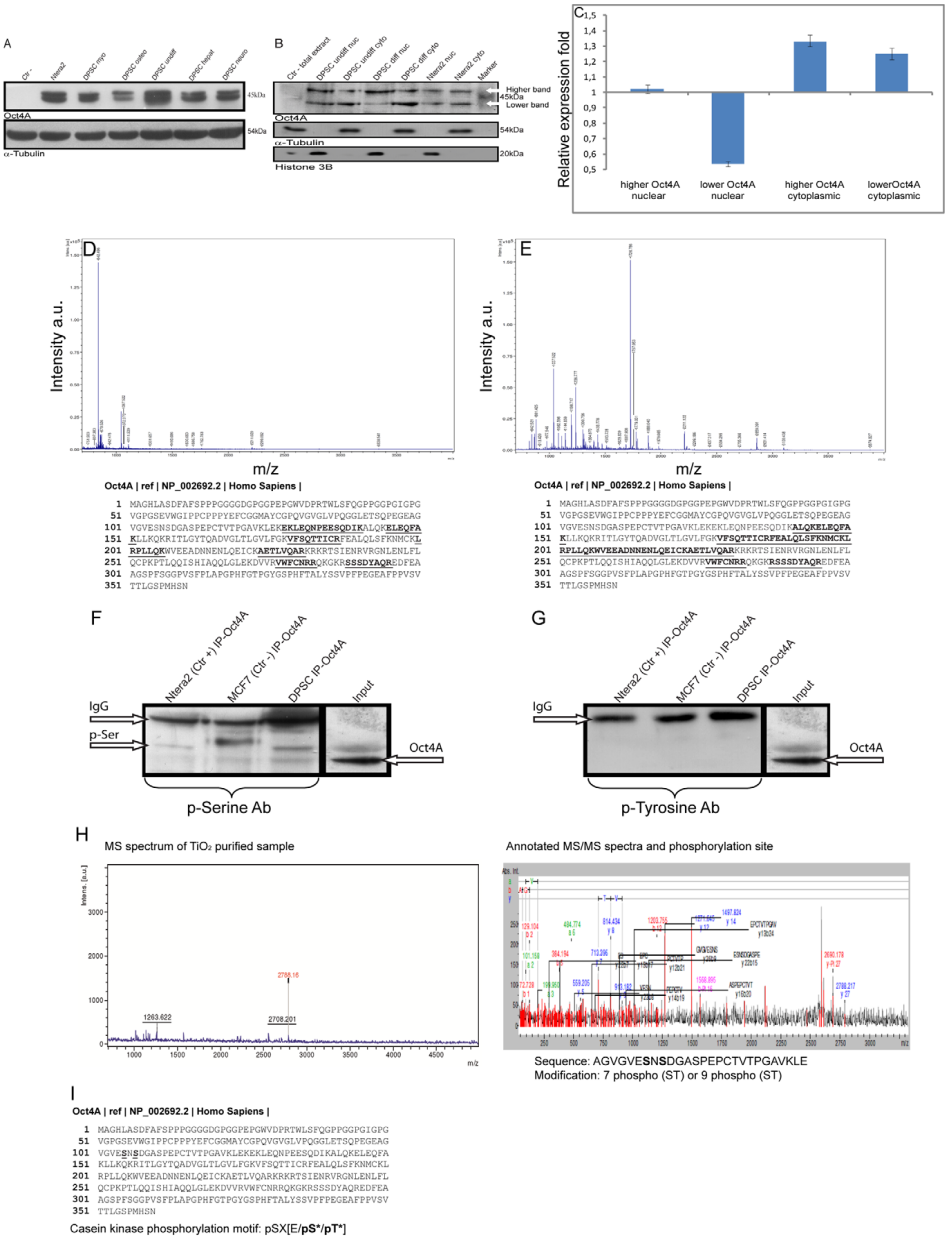
Oct4A expression, identification and phosphor analysis. (A) Undifferentiated, differentiated DPSC Ntera2 and MCF7 whole cell extracts were tested for Oct4A expression: Lane 1: Ctr – (MCF7); Lane 2: Ntera2; Lane 3: myo-differentiated DPSC; Lane 4: osteo-differentiated DPSC; Lane 5: undifferentiated DPSC; Lane 6: hepato-differentiated DPSC, Lane 7: neuro-differentiated DPSC. Anti α-tubulin antibody was used to demonstrate comparable protein loading. (B) Undifferentiated, osteo-differentiated DPSC and Ntera2, nuclear and cytoplasmic extracts, and MCF7 whole cell extract, were tested for Oct4A expression. Lane 1: Ctr – (MCF7) whole cell lysate; Lane 2: Undifferentiated DPSC nuclear extracts; Lane 3: undifferentiated DPSC cytoplasmic extracts; Lane 4: osteo-differentiated DPSC nuclear extracts; Lane 5: osteo-differentiated DPSC cytoplasmic extracts; Lane 6: Ntera2 nuclear extracts; Lane 7: Ntera2 cytoplasmic extracts; Lane 8: MW marker. Anti α-tubulin and histone H3 antibodies were used to demonstrate comparable protein loading. (C) Graph evidences DPSC osteo-differentiated over undifferentiated expression ratio for higher Oct4A nuclear band, lower Oct4A nuclear band, higher Oct4A cytoplasmic band, lower Oct4A cytoplasmic band. X-axis shows relative expression fold, Y-axis tested markers. Data are expressed as mean ratio ± SD of three independent experiments and were normalized using α-Tubulin, and histone H3, (adjusted *P*<0.05). (D, E) Oct4A immunoprecipitated samples, derived from undifferentiated DPSC, were run on SDS/PAGE and the relative bands were excised from gel and identified using mass spectrometry analysis. (D) Peptides used for the higher band identification by MS/MS sequencing are shown in bold highlighted in the sequence and relative m/z graph. (E) Peptides used for the lower band identification by MS/MS sequencing are shown in bold in the sequence and relative m/z graph. X-axis shows intensity a.u. Y-axis m/z. (F) Ntera2, MCF7 and undifferentiated DPSC samples, subjected to immunoprecipitation using anti-Oct4A, were reacted with anti-phospho serine antibodies; Lane 1: Ntera2 IP-Oct4A; Lane 2: MCF7 IP-Oct4A; Lane 3: undifferentiated DPSC IP-Oct4A; Lane 4: Input. (G) Ntera2, MCF7 and undifferentiated DPSC samples, subjected to immunoprecipitation using anti-Oct4A, were reacted with anti-phospho tyrosine antibodies; Lane 1: Ntera2 IP-Oct4A; Lane 2: Ctr -IP-Oct4A; Lane 3: DPSC IP-Oct4A; Lane 4: Input. (H) MS spectrum of TiO_2_ purified sample and annotated MS/MS spectra for Oct4A higher band, derived from undifferentiated DPSC, confirm the presence of a phosphorylation site in Oct4A protein. X-axis shows intensity Y-axis m/z. Bold highlighted amino-acid residues evidence where phosphorylation has been supposed in MS analyzed peptides. (I) Bold highlighted amino-acid residues evidence where phosphorylation has been supposed in Oct4A reference sequence NP_002692.2. CK-II consensus sequence: E, glutamic acid; S, serine; T, threonine; X, any amino acid, and * indicates the residue that has to be phosphorylated already for the enzyme to recognize the motif.

In the attempt to explain the high Oct4A expression rate and acquire new evidences about its sub-cellular localization afterward differentiation; we performed WB, extending electrophoresis running time, loading both whole cell extracts, cytoplasmic and nuclear extracts of undifferentiated, differentiated DPSC, Ntera2 and MCF7 cells. Results indicated that Oct4A protein can be resolved in two distinct bands for whole extracts (Fig. 8A), as well as for nuclear and cytoplasmic extracts (Fig. 8B) of undifferentiated, differentiated DPSC and Ntera2 cells. Nuclear and cytoplasmic bands evidenced in WB were then quantified by densitometry and the resulting data (Fig. 8C), reported as differentiated over undifferentiated DPSC expression ratio, were: higher Oct4A nuclear band (1.02±0.027) fold, lower Oct4A nuclear band (0.53±0.017) fold, higher Oct4A cytoplasmic band (1.32±0.037) fold, lower Oct4A cytoplasmic band (1.25±0.019) fold (*P*<0.05).

To confirm Oct4A identity, whole cell extracts of undifferentiated DPSC and Ntera2 were subjected to immunoprecipitation using anti Oct4A antibody, then the immunocomplexes were subjected to WB. Separated proteins were evidenced by MS compatible silver staining and the bands corresponding to those previously obtained, which we supposed to be Oct4A, were excised and subjected to protein identification by MS. Protein identification confirmed that both proteins correspond to Oct4A, as evidenced by relative m/z graphs and by Oct4A sequence matching (bold underlined peptides), respectively for higher (Fig. 8D) and lower (Fig. 8E) band.

Based on studies describing post-translational modifications of Oct4 [2], we hypothesized that the higher Oct4A band might represent one or more post-translational modifications (PTM) and specifically phosphorylations. For this reason Oct4A protein sequence was analyzed using PhosphoMotif Finder, www.hprd.org, and the results revealed 35 serine and 10 tyrosine kinase/phosphatase motifs. Therefore, taking into consideration these results, undifferentiated DPSC samples were immunoprecipitated, using anti Oct4A antibody, and were reacted with anti-phospho serine and anti-phospho tyrosine antibodies, to evidence the presence of one or more phosphorylation sites. Results showed that Oct4A immunoprecipitated samples possessed one or more phosphorylated serine (Fig 8F), instead we were not able to detect tyrosine phosphorylated residues (Fig. 8G).

To confirm Oct4A post-translational modification (PTM) Oct4A higher band, derived from undifferentiated DPSC, was extracted from the gel and was subjected to phospho-analysis using MS. MS spectrum of TiO_2_ purified sample and MS/MS annotated spectra demonstrated the presence of one possible phosphorylation site (Fig. 8H), which corresponds to Oct4A serine residue S 105 or S 107 (Fig. 8I). Unfortunately from the data it was not possible to establish whether the phosphorylation was exactly placed, however our previous phospho motif analysis evidenced that S 105 is a putative Casein Kinase II (CK-II) phosphorylation site (Fig. 8I), and phospho-motif analysis suggested that serine 107 has to be phosphorylated already for the enzyme to recognize the motif and phosphorylate serine 105.

## Discussion

There are many investigations describing the role of human DPSC for tissue regeneration, advancing their therapeutic relevance as a valuable stem cell source [14]–[16], [19]. DPSC are commonly cultured in FBS, which poses risk of transferring infections and induction of immune reactions upon transplantation. HS has been considered to be a safer alternative excluding the transfer of animal derived infections and related immunogenic reactions and our data evidence that low HS percentages support the isolation of DPSC, showing a well-defined stemness related phenotype and multilineage differentiation properties.

The aim of this study was to establish the role played by the ES core transcription factor, which includes c-Myc, Klf4, Nanog, and Oct4, as well as that of the stemness related CDs in adult stem cells. With this intent we investigated the expression pattern of these markers both in undifferentiated and fully differentiated DPSC.

It has been previously reported that stem cell differentiation is directly correlated with stemness loss, and this has been demonstrated evaluating the newly or up-regulated expression of the differentiation markers and by the absence or down-regulation of the stemness related markers [16]–[20], [31], [32]. Even if there are discrepancies between messenger and protein expression for Oct4B and c-Myc, our results confirm that afterward DPSC differentiation the stemness markers Oct4A, Klf4, Nanog, c-Myc, Oct4B as well as CD10, CD29, CD117, SSEA4, are down-regulated [31], [49]–[57]. Instead tissue specific differentiation markers are in general up-regulated after differentiation; in the same manner SSEA1 (CD15) increases after differentiation, as previously reported by Draper et al [57].

These results indicate a similarity, at least as regards these markers, between embryonic and adult stem cells [1]–[6], [16]–[20], [31], [32], [45], moreover confirm that the signaling and regulatory events affecting cell adhesion and cell membrane proteins are also involved in stemness [55], [57].

Over-expressing different combinations of three transcription factors, chosen among Klf4, Oct4, c-Myc and Sox2, causes the reprogrammation of somatic cells into a more primitive Induced Pluripotent stem cells (IPs) [51], [58]–[62], but induction was not observed for the three factor combinations that did not include Oct4 [49].

It is well established that stemness in ES cells is under the control of Oct4 [1]–[4], [49], [52], [62], in particular Niwa et al. [3] reported a direct correlation between Oct4 expression and stemness maintenance, evidencing that the Oct4 expression level drops below ±50% of normal levels when cells lose their pluripotency.

Subcellular compartimentalization of transcriptions factors is an important mechanism to regulate their activity. Many transcriptions factors localize to the cytoplasm in their basal, unstimulated state, needing to be activated and imported into nucleus to initiate the expression of their target genes, others, as Oct4A in stem cells, are located mainly in the nucleus where initiates the expression of their target genes; however both types are exported from the nucleus for their recycling and/or regulation [63]–[66]. It has been also demonstrated in different cell types, as well as in ES cells, the identification of a phosphorylation mediated mechanism, which permits nuclear/cytoplasmic translocation degradation and consequent cell differentiation [64]–[66]. In this research we provide evidence that nuclear Oct4A is down-regulated to about 50% during DPSC differentiation, as well as that a post-translationally modified Oct4A form increases in cytoplasm afterward the acquisition of the differentiated phenotype. Moreover, MS permitted us to suppose a CK-II-dependent serine phosphorylation site at residue 105 or 107 in Oct4A protein, and phospho-motif analysis suggested that serine 107 has to be phosphorylated already for the enzyme to recognize the motif and phosphorylate serine 105.

CK-II is a ubiquitous Ser/Thr protein kinase that plays a central role in the regulation of a variety of cellular processes, and has been found to facilitate phosphorylation of nuclear proteins [65] and mediate their nuclear/cytoplasmic shuttling [66] *(i.e. via* CRM1-dependent mechanism [67]). Oct4 m-RNA and protein disappear relatively quickly following differentiation [1]–[4], but at the same time have been also noted their re-appearance [5], [6] suggesting that a critical amount of Oct4 is required to maintain stemness [3]. Taking into consideration these observations, this study confirms Oct4A importance in stemness maintenance even in adult stem cells, and suggests that a CK-II-dependent phosphorylation mechanism could be involved in its nucleo/cytoplasmic shuttling, by which could balance stemness versus differentiation in DPSC. Considering the Oct4A importance, we think that this could not be the sole mechanism involved in the regulation of its nuclear amount and we also suppose the presence of a redundant control mechanism, both for Oct4A as well as for the other core transcription factors, as already reported for Klf4 [64]. In conclusion understanding and controlling these mechanisms may be of great importance for stemness maintenance and stem cells clinical use, as well as for cancer research.
